# Predicting Treatment Failure for Initiators of Hepatitis C Virus Treatment in the era of Direct-Acting Antiviral Therapy

**DOI:** 10.3389/fphar.2020.551500

**Published:** 2020-11-13

**Authors:** Nadia A. Nabulsi, Michelle T. Martin, Lisa K. Sharp, David E. Koren, Robyn Teply, Autumn Zuckerman, Todd A. Lee

**Affiliations:** ^1^ University of Illinois at Chicago College of Pharmacy, Chicago, IL, United States; ^2^ University of Illinois Hospital and Health Sciences System, Chicago, IL, United States; ^3^ Temple University Hospital, Philadelphia, PA, United States; ^4^ Creighton University School of Pharmacy and Health Professions, Omaha, NE, United States; ^5^ Vanderbilt University Medical Center – Specialty Pharmacy Services, Nashville, TN, United States

**Keywords:** hepatitis C virus, sustained virologic response, treatment failure, prediction model, direct-acting antivirals

## Abstract

**Introduction:** Hepatitis C virus (HCV), the leading cause of advanced liver disease, has enormous economic burden. Identification of patients at risk of treatment failure could lead to interventions that improve cure rates.

**Objectives:** Our goal was to develop and evaluate a prediction model for HCV treatment failure.

**Methods:** We analyzed HCV patients initiating direct-acting antiviral therapy at four United States institutions. Treatment failure was determined by lack of sustained virologic response (SVR) 12 weeks after treatment completion. From 20 patient-level variables collected before treatment initiation, we identified a subset associated with treatment failure in bivariate analyses. In a derivation set, separate predictive models were developed from 100 bootstrap samples using logistic regression. From the 100 models, variables were ranked by frequency of selection as predictors to create four final candidate models, using cutoffs of ≥80%, ≥50%, ≥40%, and all variables. In a validation set, predictive performance was compared across models using area under the receiver operating characteristic curve.

**Results:** In 1,253 HCV patients, overall SVR rate was 86.1% (95% CI = 84.1%, 88.0%). The AUCs of the four final candidate models were: ≥80% = 0.576; ≥50% = 0.605; ≥40% = 0.684; all = 0.681. The best performing model (≥40%) had significantly better predictive ability than the ≥50% (*p* = 0.03) and ≥80% models (*p* = 0.02). Strongest predictors of treatment failure were older age, history of hepatocellular carcinoma, and private (vs. government) insurance.

**Conclusion:** This study highlighted baseline factors associated with HCV treatment failure. Treatment failure prediction may facilitate development of data-driven clinical tools to identify patients who would benefit from interventions to improve SVR rates.

## Introduction

Hepatitis C virus (HCV) is the leading cause of advanced liver disease and an enormous economic burden that caused an estimated $6.5 billion in United States healthcare costs in 2011 (Razavi et al., 2013). If untreated, HCV can lead to liver fibrosis, cirrhosis, hepatocellular carcinoma (HCC), and death. The Centers for Disease Control and Prevention (CDC) reported over 15,000 deaths related to HCV in 2018, and about 2.4 million cases of chronic HCV in the United States from 2013 to 2016 (Edlin et al., 2015; Hofmeister et al., 2019; Centers for Disease Control and Prevention, 2020). Over half of HCV-infected patients in the United States are unaware of their infection, which may lead to additional incident cases (Hagan et al., 2006; Smith et al., 2012; Chhatwal et al., 2016).

Direct-acting antiviral (DAA) treatment offers HCV cure rates of over 95% (Burstow et al., 2017; Kish et al., 2017). However, the high cost of DAA therapy coupled with the large number of patients eligible for treatment could significantly impact payer budgets (Brennan and Shrank, 2014; Chhatwal et al., 2015a). Since these highly effective medications are not universally affordable throughout the world, there is a need to develop cost-effective strategies such as resource- and value-based management of HCV patients with the aim of obtaining the most benefit for the least expenditure (Chhatwal et al., 2015b; Stepanova and Younossi, 2017). Consequently, payer barriers prevent many patients from medication access. Gowda and colleagues reported an overall insurance denial rate of 36% between 2016 and 2017, with the denial rate as high as 52% for commercially-insured patients (Gowda et al., 2018). Additionally, in some states, Medicaid only covers treatment for patients with advanced hepatic fibrosis, meaning patients must wait until their liver damage significantly progresses before they can undergo HCV treatment; yet coverage is not guaranteed even then (Lo Re et al., 2016). Some state Medicaid sites also limit treatment access based on provider specialty and patient history of alcohol and/or substance use (Barua et al., 2015). These barriers highlight the current emphasis on payer limitations for costly regimens.

In line with efforts to increase cost-effectiveness, practical risk assessment tools to identify patients at high risk of treatment failure [including early discontinuation, loss to follow-up, and failure to achieve sustained virologic response (SVR)] could lead to interventions that improve SVR rates without imposing additional barriers to treatment access. Currently, validated tools to identify patients at high risk of HCV treatment failure do not exist and although barriers to HCV treatment initiation have been explored, factors associated with unsuccessful treatment completion have not been elucidated (Morrill et al., 2005; Kattakuzhy et al., 2017). In light of evidence that a large loss of patients throughout the HCV treatment cascade occurs after treatment initiation, early identification and intervention of these patients who initiate but fail treatment may have significant positive economic and public health impact (DeBose-Scarlett et al., 2018). Therefore, in alignment with a broader movement toward cost-effective personalized HCV therapy and identification of valid predictors of response for a given patient (Ferenci, 2004; Ochi et al., 2012; Beinhardt et al., 2013; Petta and Craxi, 2013; Andriulli et al., 2014; Ansaldi et al., 2014; Mathes et al., 2014; Petta et al., 2014; Thompson et al., 2014; Iannazzo et al., 2015; Backus et al., 2016; Jansen et al., 2017; Su et al., 2017; Kouris et al., 2018), our aim was to develop and evaluate a prediction model of treatment failure in patients initiating DAA therapy using demographic and clinical characteristics measured before treatment initiation.

## Methods

### Study Design

We conducted a secondary analysis of data collected from a multi-site cohort of HCV patients initiating DAA therapy. The original study was an observational retrospective cohort study utilizing electronic medical record (EMR) data from four United States institutions (Koren et al., 2019). The four sites were the University of Illinois Hospital and Health Sciences System (UI Health), Vanderbilt University Medical Center (VUMC), Temple University Hospital (TUH), and Creighton University (CU). The study was reviewed and approved by the institutional review board at each of the participating institutions.

### Study Population

Adult patients (18 years of age or older) initiating a dual or triple all-oral DAA HCV treatment regimen between January 1, 2014 and March 12, 2018 treated within a pharmacist-driven interdisciplinary model at each institution were identified for inclusion. Patients were excluded if their anticipated date of 12 weeks post-treatment completion was after September 7, 2018.

### Study Protocol

After patients were identified for inclusion in the study, data were extracted from EMRs at each of the institutions. Baseline data were extracted from the 12-month period prior to treatment initiation. For information reported multiple times during the baseline period, the value closest to the treatment initiation date was used. In our aim to develop a predictive model of treatment failure for patients initiating treatment, we only considered baseline data for inclusion in the prediction model.

Baseline data were collected for several potential candidate predictor variables. Categorically-defined variables included age (18–64 years, 65 years or older), sex, body mass index (BMI) (less than 30, 30 or higher), ethnicity, insurance type, HCV genotype, HCV treatment history, fibrosis stage, Child-Turcotte-Pugh class in patients with cirrhosis, hepatitis B virus and/or HIV coinfection, solid organ transplant history, dialysis status, comorbid diabetes or psychiatric illness, and presence of drug-drug interactions (DDIs) with intended DAA regimen. Insurance type was categorized as Medicaid (joint federal and state program that provides health coverage to low-income individuals and individuals with disabilities) (Centers for Medicare and Medicaid Services, 2020a), Medicare (federal health insurance program for individuals who are 65 years or older, individuals who are younger than 65 years with disabilities, and individuals with end-stage renal disease) (Centers for Medicare and Medicaid Services, 2020b), private (or commercial) insurance (health insurance that is employer-sponsored, privately purchased, or acquired through the Health Insurance Marketplace under the Affordable Care Act) (Blumenthal et al., 2015), or none/unknown. Patient-reported history of and/or current alcohol use, illicit substance use, and intravenous drug use (IVDU) was also determined from chart documentation. Continuous variables included HCV RNA levels, baseline alanine aminotransferase and aspartate aminotransferase levels, serum creatinine, platelets, total bilirubin, and albumin.

### Primary Outcome Measure

The primary outcome to be predicted was treatment failure, defined as early discontinuation, lost to follow-up (LTFU), or failure to achieve SVR. Patients were considered to have discontinued treatment early if they did not complete their initially prescribed length of treatment for any reason, including provider instruction, self-discontinuation, or death. Patients were considered LTFU if they did not have SVR data available at a minimum of 12 weeks after the completion of therapy for any reason, including death. Failure to achieve SVR was defined as a detectable HCV RNA polymerase chain reaction test result at least 12 weeks after HCV treatment completion.

### Statistical Analysis

All analyses were conducted using SAS 9.4 software (SAS Institute, Cary, NC, United States). The rates of treatment success and failure among the entire sample were determined and expressed as frequencies and proportions. To develop predictive models, we applied bootstrap statistical methods as described by Austin and Tu (Austin and Tu, 2004). Among the baseline variables collected, 20 categorical variables were selected *a priori* based on pharmacists’ clinical experience for assessment as predictors of treatment failure.

After identifying 20 candidate predictor variables, we tested variables for collinearity using Pearson correlation coefficients and examined prevalence in the overall sample to inform the first stage of variable elimination. For variables that displayed collinearity, investigators met to come to a consensus regarding which of the two correlated variables should be excluded. Variables prevalent in less than 3% of the sample were also removed from the set of candidate predictors to avoid sparse data bias.

The cohort was randomly divided into a derivation (two thirds of the original dataset) and validation (one third of the original dataset) set while maintaining the original treatment failure rate in each set. Model development was completed using the derivation set.

In the derivation set, descriptive analyses were completed on all 20 candidate predictor variables to define the patient sample using frequencies and proportions. We assessed associations between each of the candidate variables and treatment failure using bivariate analyses and chi-squared tests of independence in the derivation set to inform the second stage of variable elimination. Variables that exhibited a significant association with treatment failure with a significance level of *p* < 0.25 were retained in the set of candidate predictors to be used in multivariable regression models (Hosmer and Lemeshow, 1989).

We generated 100 samples from the derivation set using nonparametric bootstrapping. Stepwise logistic regression adjusted for a cluster effect within each participating institution was applied to develop a parsimonious predictive model of treatment failure within each bootstrap sample (Wang and Shin, 2011). We used *p* = 0.05 for the criterion for entry into the model and for staying in the model. Subsequently, we determined the number of times each variable was selected as a predictor across all 100 bootstrap samples. These frequencies of variable selection were used to inform the creation of four candidate models for predicting treatment failure. The predictive models were comprised of variables that were selected through stepwise logistic regression in at least 80, 50, and 40% of the bootstrap samples. A final predictive model was developed using all candidate predictors remaining after the second stage of variable elimination.

Once the four candidate models were established, we determined the predicted probability of treatment failure for each patient using the validation set. The goodness-of-fit and predictive performance were assessed in the validation set using the Hosmer-Lemeshow (HL) goodness-of-fit test and area under the receiver operating characteristic (ROC) curve (AUC), respectively. Subsequently, the AUC of each candidate model was compared to chance (AUC = 0.5) using ROC contrast estimation in the validation set. The AUC of the best performing model was also compared to every other model to determine if its predictive ability was significantly better than the alternative candidate models.

In analyzing the robustness of the results, we determined the distribution of beta coefficients across all bootstrap samples in which the variable was selected. Bootstrap regression coefficients that are either all positive or all negative indicate model stability.

While the first stage of variable elimination attempted to eliminate multicollinearity, we also compared all pairwise combinations of the candidate predictors that were included in the model-building process (i.e., those variables remaining after the second stage of variable elimination) to identify possible concerns with multicollinearity. If a pair of variables appeared to mutually exclude each other (indicating multicollinearity) and if each variable were only included in <50% of the bootstrap models, then one of the two variables was removed from the set of candidate predictors and the model-development process was repeated.

## Results

Our overall sample was comprised of 1,253 patients who initiated dual or triple all-oral DAA HCV treatment between January 1, 2014 and March 12, 2018, including 782 (62.4%) patients from UI Health, 279 (22.3%) from VUMC, 138 (11.0%) from TUH, and 54 (4.3%) from CU. Patients were primarily ethnic minority (69.1%) and male (63.9%), with a mean age of 57.4 years (SD = 10.1). In the sample, 1,079 (86.1%) patients achieved SVR and were identified as treatment successes while 174 (13.9%) were identified as treatment failures. Among the 174 patients who failed treatment, 24 (13.8%) discontinued treatment early, 95 (54.6%) were LTFU, and 55 (31.6%) failed to achieve SVR.

The 20 baseline candidate predictor variables selected initially included 1) sex; 2) ethnicity; 3) age; 4) insurance type; 5) history of alcohol use; 6) history of IVDU; 7) history of other illicit substance use; 8) past HCV treatments; 9) presence of DDIs; 10) genotype; 11) opioid substitution therapy (OST); 12) post-transplant immunosuppression agents; 13) solid organ transplantation; 14) HCC; 15) BMI, 16) diabetes, 17) HIV coinfection, 18) cirrhosis, 19) dialysis, and 20) psychiatric illness. From this list, dialysis and the use of post-transplant immunosuppression agents were removed in the first stage of variable elimination due to prevalence in less than 3% of the sample and collinearity with history of solid organ transplantation, respectively. Chi-squared tests revealed significant differences (*p* < 0.05) in all 18 remaining candidate predictors across institutions, supporting the use of a clustered approach in regression analyses.

After splitting the original dataset while stratifying by SVR rate, the derivation set contained 837 observations with 117 (14.0%) treatment failures and the validation set contained 416 observations with 57 (13.7%) treatment failures. Table 1 presents characteristics of the sample within the derivation set and bivariate associations between the 20 original candidate predictor variables and treatment failure. Table 1 also indicates which variables were removed in the first and second stages of variable elimination.

**TABLE 1:** Sample characteristics as described by original baseline candidate predictor variables and associations with treatment failure in derivation set.

		Treatment failure	Treatment success	*p*-value
		n = 117	n = 720	
Candidate predictor variable		n (%)	n (%)	
Final candidate predictor variables				
Age ≥65 years		17 (14.5)	174 (24.2)	0.021
Insurance type	Medicaid	39 (33.3)	259 (36.0)	0.237
	Medicare	34 (29.1)	219 (30.4)	
	Private (commercial) insurance	29 (24.8)	187 (26.0)	
	None/Unknown	15 (12.8)	52 (7.2)	
History of IVDU (last 6–12 months)		11 (9.4)	38 (5.3)	0.078
History of alcohol use (last 6–12 months)		44 (37.6)	229 (31.8)	0.214
HIV Coinfection		11 (9.4)	128 (17.8)	0.024
Current use of OST		11 (9.4)	30 (4.2)	0.015
Presence of DDIs		46 (39.3)	341 (47.4)	0.106
History of HCC		10 (8.5)	28 (3.9)	0.0248
Received prior HCV treatment (experienced)		29 (24.8)	133 (18.5)	0.109
Cirrhosis		59 (50.4)	290 (40.3)	0.039
History of solid organ transplantation		5 (4.3)	55 (7.6)	0.191
Variables removed in stage 1				
Current use of post-transplant immunosuppression agents		5 (4.3)	54 (7.5)	0.206
Current dialysis		1 (0.9)	22 (3.1)	0.233
Variables removed in stage 2				
Male		72 (61.5)	467 (64.9)	0.486
Ethnicity	African American/Black	58 (49.6)	390 (54.2)	0.694
	Caucasian	41 (35.0)	222 (30.8)	
	Hispanic	14 (12.0)	91 (12.6)	
	Other/Unknown	4 (3.4)	17 (2.4)	
History of other illicit substance use (last 6–12 months)		16 (13.7)	109 (15.1)	0.680
Obesity (BMI ≥30)		48 (41.0)	271 (37.6)	0.491
Diabetes		30 (25.6)	179 (24.9)	0.857
Psychiatric illness		40 (34.2)	225 (31.3)	0.526
Genotype 1		101 (86.3)	639 (88.8)	0.447

DDIs, Drug-drug interactions; HCC, Hepatocellular carcinoma; IVDU, Intravenous drug use; OST, Opioid substitution therapy.

**FIGURE 1:**
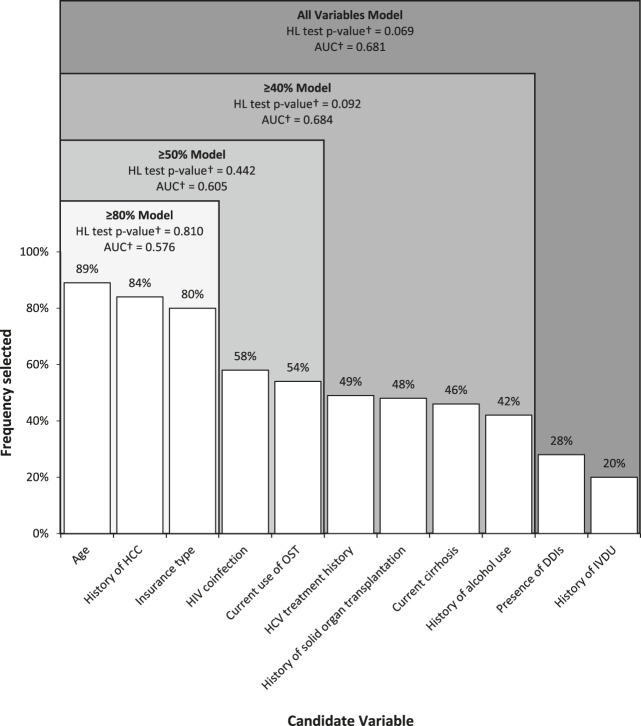
Frequency of selection of candidate variables as predictors using stepwise logistic regression in 100 bootstrap samples drawn from derivation set and performance of resulting predictive models in validation set. AUC, area under the receiver operating characteristic curve; DDIs, Drug-drug interactions; HL, Hosmer-Lemeshow; HCC, Hepatocellular carcinoma; IVDU, Intravenous drug use; OST, Opioid substitution therapy. Note: Cutoffs indicate variables included in four separate predictive models developed from variables selected in ≥80%, ≥50%, and ≥40% of bootstrap samples and all variables † HL goodness-of-fit test p-value and AUC determined from testing models in the validation set.

The frequency of selection of each variable in the 100 bootstrap samples from the derivation set are shown in Figure 1. Age, history of HCC, and insurance type were selected most often, with selection frequencies of 89, 84, and 80% in the bootstrap samples, respectively.

Frequency selection thresholds were applied to create four separate candidate predictive models that included variables that were selected in ≥80, ≥50, and ≥40% of bootstrap samples and all variables. Each model’s variables, goodness-of-fit, and predictive performance (determined from testing models in the validation set) are displayed in Figure 1. As indicated in Figure 1, the HL goodness-of-fit test *p*-values corresponding to all four candidate models were >0.05, indicating that the models fit the data well. Also shown in Figure 1, the highest AUC corresponded to the ≥40% model, indicating that the model from this threshold had the highest predictive ability of the four models.

Three of the four candidate models were significantly better than chance in their predictive ability to discriminate between treatment success and treatment failure, with *p*-values of 0.058 for the ≥80% model, 0.009 for the ≥50% model, and <0.0001 for the ≥40% model and the all variable model. When comparing the model with the highest predictive ability, the ≥40% model, to the other three candidate models, it was observed that this model had significantly better predictive ability than the ≥80% model (*p* = 0.012) and the ≥50% model (*p* = 0.029), but did not have significantly better predictive ability than the all variables model (*p* = 0.723).

**FIGURE 2:**
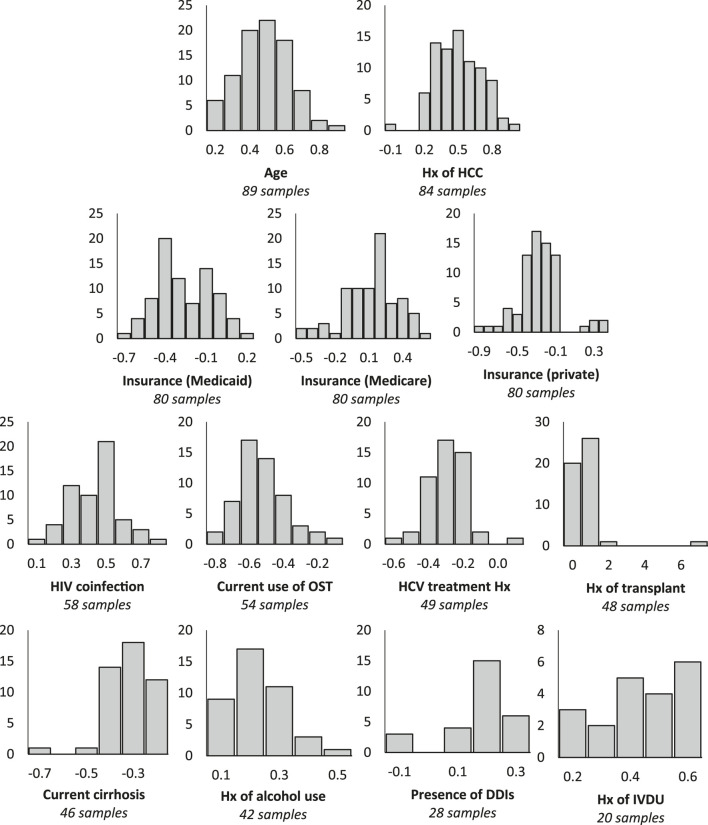
Robustness analysis of bootstrap beta coefficient distributions across samples in which variable was selected as a significant predictor of treatment failure in derivation set. DDIs: Drug-drug interactions; HCC: Hepatocellular carcinoma; HIV: Human immunodeficiency virus; Hx: History; IVDU: Intravenous drug use; OST: Opioid substitution therapy. Horizontal axis: β coefficient; Vertical axis: Frequency; Comparisons: Age - Less than 65 vs. 65 or older; Hx of HCC - Yes vs. no; Insurance (Medicaid) - Medicaid vs. none/unknown; Insurance (Medicare) - Medicare vs. none/unknown; Insurance (private) - Private vs. none/unknown; HIV coinfection - No vs. yes; Current use of OST - No vs. yes; HCV treatment Hx - Naïve vs. experienced; Hx of transplant - No vs. yes; Current cirrhosis - No vs. yes; Hx of alcohol use - Yes vs. no; Presence of DDIs - No vs. yes; Hx of IVDU - Yes vs. no.

Robustness analyses (Figure 2) revealed that the majority of bootstrap beta coefficient distributions did not cross the null value of 0, indicating general consistency of the beta coefficients across bootstrap replications and supporting model stability. pairwise examination between candidate predictor variables (Table 2) was not indicative of any significant issues related to multicollinearity.

**TABLE 2:** Pairwise examination of multicollinearity for categorical candidate predictor variables.

Variable	Percentage of models with variable^a^	Age	History of HCC	Insurance type	HIV coinfection	Current use of OST	HCV treatment hx	Hx of solid organ transplantation	Current cirrhosis	Hx of alcohol use (last 6–12 months)	Presence of DDIs	Hx of IVDU (last 6–12 months)
Age	89	100	83	82	58	54	51	47	47	45	28	19
History of HCC	84	88	100	77	56	50	48	55	42	42	27	21
Insurance type	80	91	81	100	56	56	54	46	50	46	29	13
HIV coinfection	58	90	81	78	100	57	50	55	50	47	17	16
Current use of OST	54	89	78	83	61	100	41	39	44	44	33	13
HCV treatment hx	49	92	82	88	59	45	100	49	51	49	20	22
Hx of solid organ transplantation	48	88	96	77	67	44	50	100	48	33	10	15
Current cirrhosis	46	91	76	87	63	52	54	50	100	52	26	17
Hx of alcohol use (last 6–12 months)	42	95	83	88	64	57	57	38	57	100	26	17
Presence of DDIs	28	89	82	82	36	64	36	18	43	39	100	32
Hx of IVDU (last 6–12 months)	20	85	90	50	45	35	55	35	40	35	45	100

DDIs, Drug-drug interactions; HCC, Hepatocellular carcinoma; Hx, History; IVDU, Intravenous drug use; mos, Months; OST, Opioid substitution therapy.

a Displays the percentage of bootstrap samples in which the candidate variable was selected as a predictor of treatment failure. The remaining columns display the percentage of bootstrap samples in which the row variable was selected as a predictor of treatment failure and the column variable was also selected as a predictor of treatment failure.

## Discussion

Using a cohort of patients treated with DAAs for their HCV, we used a rigorous approach to develop and evaluate the performance of a prediction model for treatment failure. The most parsimonious predictive model with the highest predictive ability included nine variables, namely age, history of HCC, insurance type, HIV coinfection, current use of OST, HCV treatment history, history of solid organ transplantation, current cirrhosis, and history of alcohol use. This model demonstrated predictive ability significantly better than models with fewer variables. However, this model displayed relatively poor ability to discriminate between treatment failures and treatment successes, as indicated by the AUC of 0.68.

Three variables were selected in 80% or more of the bootstrap models (age, history of HCC, and insurance type), indicating robust associations with treatment failure. There is some supporting evidence of associations between these variables and SVR rates. Older age had been shown to be associated with lower SVR rates in the interferon era, possibly due to more frequent adverse effects among older patients and resulting treatment discontinuation (Mathes et al., 2014; Sato et al., 2015; Tsui et al., 2008). Reid et al. speculate that lower SVR rates in the older population could be due to an increased severity of HCV-associated liver disease caused by longer duration of HCV infection or aging-related mechanisms (Reid et al., 2017). Studies have also found that the presence of HCC at the initiation of DAA therapy is significantly associated with treatment failure in patients with HCV, for reasons that are not entirely clear (Prenner et al., 2017; Konjeti and John, 2018; Kushner et al., 2018). Finally, insurance status may be a significant predictor of treatment failure as patients who are denied HCV therapy have a higher risk of developing more severe HCV-related symptoms and complications, such as cirrhosis and HCC, making them more difficult to cure (DeBose-Scarlett et al., 2018). Given that the incidence of absolute denial of DAA therapy has been found to be substantially higher among patients with private insurance vs. Medicaid or Medicare (Gowda et al., 2018), it is possible that the commercially-insured patients in our sample encountered more barriers to accessing HCV treatment. This may partially explain why our results indicate that private insurance is a strong predictor of treatment failure. Though many United States states have eased prescribing restrictions in recent years possibly in response to decreases in HCV therapy prices (AASLD and IDSA HCV Guidance Panel, 2020), the health and economic impacts associated with HCV treatment restrictions enforced by insurers, particularly commercial insurers, should continue to be investigated as HCV elimination goals are pursued (Chidi et al., 2016; Behzadifar et al., 2019; Goodyear et al., 2020; Liu et al., 2020; Price, 2020; Saeed et al., 2020).

Prior studies have attempted to identify significant predictors of treatment failure (Ferenci, 2004; Ochi et al., 2012; Beinhardt et al., 2013; Petta and Craxi, 2013; Andriulli et al., 2014; Ansaldi et al., 2014; Mathes et al., 2014; Thompson et al., 2014; Backus et al., 2016; Jansen et al., 2017; Su et al., 2017; Kouris et al., 2018). However, most examined older interferon treatment options that are no longer prescribed for HCV (Ferenci, 2004; Ochi et al., 2012; Beinhardt et al., 2013; Petta and Craxi, 2013; Andriulli et al., 2014; Ansaldi et al., 2014; Mathes et al., 2014; Thompson et al., 2014). Two recently published studies examined predictors of DAA treatment failure, but applied less robust statistical methods with relatively small samples sizes (Jansen et al., 2017; Kouris et al., 2018). The first study, a retrospective case-control analysis in adult veterans treated with ledipasvir/sofosbuvir, only identified elevated pretreatment creatinine clearance as a potential predictor of treatment failure (Jansen et al., 2017). This study was limited by a small sample size of 156 patients, with only 12 treatment failures, and a high exclusion rate. The second study, a retrospective cohort analysis of Medicaid members treated with ledipasvir/sofosbuvir for HCV genotype 1 infection, concluded that none of the clinical and demographic variables assessed (sex, history of prior treatment failure, cirrhosis, substance use disorder, HIV-coinfection, and concomitant use of interacting medications) were significantly associated with treatment failure (Kouris et al., 2018). However, this analysis excluded patients who did not complete treatment or were LTFU and therefore had a small number of patients who failed treatment, which limited the ability to identify statistically significant associations.

Additional relevant studies by Backus et al. and Su et al. among veterans found that Black race and Hispanic ethnicity were significant independent predictors of treatment failure among patients who initiated DAA regimens, for reasons that remain unclear (Backus et al., 2016; Su et al., 2017). Although we had an ethnically diverse sample, we did not detect an association between race/ethnicity and treatment failure. The institutions included in our analysis have extensive experience caring for ethnic minority patients and addressing ethnic health disparities, which may contribute to the lack of an observed effect. However, ethnicity should continue to be evaluated as a potential predictor of treatment failure, as ethnic minorities are often underrepresented in clinical trials and face unique barriers to healthcare access.

Since our three combined outcomes (i.e., early discontinuation, LTFU, or failure to achieve SVR) all preclude a measured SVR, which is the goal of HCV therapy, we chose to measure them collectively as treatment failures rather than analyze each outcome individually. Notably, the majority of treatment failures were patients who were LTFU or discontinued treatment, while only 55 patients (4.4% of the full cohort) truly did not achieve SVR as determined by post-treatment week 12 labs. Excluding patients who were LTFU or discontinued treatment early would not provide a complete assessment of the undesirable outcomes of real-world clinical practice. There is a need to focus on retaining patients throughout the course of treatment and ensuring medication, lab, and appointment adherence rather than improving the effectiveness of available therapies. This is particularly true in the United States, where prohibitive copayments may be a significant barrier to treatment access and adherence (DeBose-Scarlett et al., 2018; Rosenthal and Graham, 2016; Sarpel et al., 2016). Early identification of patients who are likely to drop out or discontinue treatment may inform interventions to maintain adherence and follow-up.

Identifying predictors of HCV treatment failure prior to the initiation of therapy is important in recognizing high-risk patients and alerting clinicians as to whether they should further intervene to address potential barriers. These efforts could ultimately provide a tool to guide additional treatment monitoring strategies, personalized interventions, and strategic allocation of resources or additional case management to more closely follow up with at-risk patients and work to avoid treatment failure. Ultimately, identification of predictors for treatment failure could help decrease health care costs for patients and the healthcare system by avoiding necessary retreatment and long-term patient and public health outcomes associated with unattained SVR.

This analysis could be expanded upon in the future by examining patients’ past patterns of behavior, such as adherence history, interaction with the healthcare system, and utilization of healthcare resources. However, including these variables may reduce the practicality of a resulting clinical risk assessment tool, as they would be more difficult to evaluate upon initial HCV treatment referral. This study could also be built upon by assessing on-treatment factors in addition to baseline factors evaluated here. For example, there is currently not an evidence-based minimum threshold of adherence recommended to achieve SVR, but it is likely a major contributor to non-achievement of SVR (Younossi et al., 2016). Additionally, since many patients are undiagnosed, untreated, and experience difficulty accessing HCV specialists, there is a need for HCV management in the primary care setting, which warrants further investigation into the association of treatment failure with clinic and provider characteristics (Vutien et al., 2017).

A strength of this study is the combination of bootstrap sampling with automated variable selection. Automatic variable selection methods alone may generate a final model that includes spurious variables that are not true predictors of the outcome. The addition of bootstrapping facilitated assessment of the strength of the evidence that a given variable is truly a predictor based on its frequency of selection among bootstrap samples. Bootstrap sampling also allowed us to conduct a robustness check to identify model instability, which cannot be easily assessed with traditional automatic variable selection methods. An additional strength is the ethnically-diverse, real-world, multi-institutional cohort that included patients under a variety of insurance plans, increasing the generalizability of the findings. The analysis also incorporated valuable data on sociodemographic and social history characteristics that are not commonly assessed in HCV treatment failure prediction models.

### Limitations

One noteworthy limitation of this study is that it involved a secondary data analysis. Therefore, not all variables that were thought to be relevant to treatment failure by clinicians and investigators were available to include in the regression model, such as past patterns of behavior. However, the candidate predictors that were examined included variables that can be quickly and easily evaluated before HCV treatment initiation, which is crucial in strengthening the usefulness and feasibility of clinical risk assessment tools (Fazel and Wolf, 2018).

A potential limitation of our statistical approach is the possible effect of multicollinearity in which correlated predictors compete for model inclusion during stepwise regression, causing neither variable to be recognized as a strong predictor (Austin and Tu, 2004). We attempted to eliminate issues related to multicollinearity through both the first stage of variable elimination and the comparison of all pairwise combinations of candidate predictors as described in the *Methods*.

Lastly, it is important to note that high copayments and the lack of a single payer system can contribute to treatment failures in the United States, which limits the generalizability of a treatment failure prediction model beyond the United States healthcare system (Rosenthal and Graham, 2016; DeBose-Scarlett et al., 2018). Other developed countries may have more affordable medicines and different cost control mechanisms, which may help to lower treatment failure rates once treatment is initiated (Zoulim et al., 2015; Ferrario et al., 2017; Kanavos et al., 2017; Andersson et al., 2020). In low- and middle-income countries, where the majority of patients with HCV reside and issues of overall affordability still prevent utilization, treatment failure prediction models may also be less relevant (Hill et al., 2014; Phelan and Cook, 2014; Andrieux-Meyer et al., 2015; Zoulim et al., 2015; Lanini et al., 2016; Rosenthal and Graham, 2016).

## Conclusion

This study identified patient characteristics that were significant predictors of HCV treatment failure, but predicting response for an individual patient remains difficult. If this type of analysis is built upon with additional candidate predictors, it may be possible to develop a simple data-driven clinical tool to identify patients at risk of treatment failure at the time of referral for HCV treatment. Personalized interventions can then be implemented to help patients attain SVR. Ultimately, validated treatment failure prediction models may help to close the gap between HCV patients who initiate treatment and those who achieve a measured SVR to assist in reducing the significant burden of HCV.

## Data Availability Statement

The raw data supporting the conclusions of this article will be made available by the authors, without undue reservation.

## Ethics Statement

The study was reviewed and approved by the institutional review boards at 1) the University of Illinois Hospital and Health Sciences System (UI Health), 2) Vanderbilt University Medical Center (VUMC), 3) Temple University Hospital (TUH), and 4) Creighton University (CU). Written informed consent for participation was not required for this study in accordance with the national legislation and the institutional requirements.

## Author Contributions

NN and TL designed the study, performed statistical analysis, and wrote and revised the manuscript. LS assisted with designing the study/analysis and with writing and revising the manuscript. DK, MM, RT, and AZ performed data collection and assisted with writing and revising the manuscript. All authors gave final approval and are accountable for the information presented.

## Funding

This study was conducted under an investigator sponsored research grant from Gilead Sciences Inc. grant number: IN-US-342-4530. The contents are solely the responsibility of the authors. The funder had no role in study design, data collection and analysis, decision to publish, or preparation of the manuscript.

## Conflicts of Interest

NN, TL, DK, MM, RT, and AZ received investigator-sponsored research funding from Gilead Sciences, Inc. DK, MM, and RT have served on advisory board(s) for Gilead. DK has served on an advisory panel for ViiV Healthcare. MM and RT have served on advisory boards and the speaker’s bureau for for AbbVie, and RT serves on the speaker’s bureau for Gilead Sciences, Inc. MM is a minor shareholder of AbbVie, Gilead, and Merck. Subsequent to submission of this manuscript, RT has taken a position at Pfizer.

The remaining author declares that the research was conducted in the absence of any commercial or financial relationships that could be construed as a potential conflict of interest.
